# Determination of a pharmacokinetic model for [^11^C]-acetate in brown adipose tissue

**DOI:** 10.1186/s13550-019-0497-6

**Published:** 2019-03-27

**Authors:** Marie Anne Richard, Denis P. Blondin, Christophe Noll, Réjean Lebel, Martin Lepage, André C. Carpentier

**Affiliations:** Faculté de médecine et des sciences de la santé, Université de Sherbrooke, 3001 12th Avenue North, Sherbrooke, QC J1H 5N4 Canada

**Keywords:** [^11^C]-acetate, Kinetic modeling, Oxidation, Brown adipose tissue

## Abstract

**Background:**

[^11^C]-acetate positron emission tomography is used to assess oxidative metabolism in various tissues including the heart, tumor, and brown adipose tissue. For brown adipose tissue, a monoexponential decay model is commonly employed. However, no systematic assessment of kinetic models has been performed to validate this model or others.

The monoexponential decay model and various compartmental models were applied to data obtained before and during brown adipose tissue activation by cold exposure in healthy men. Quality of fit was assessed visually and by analysis of residuals, including the Akaike information criterion. Stability and accuracy of compartmental models were further assessed through simulations, along with sensitivity and identifiability of kinetic parameters.

**Results:**

Differences were noted in the arterial input function between the warm and cold conditions. These differences are not taken into account by the monoexponential decay model. They are accounted for by compartmental models, but most models proved too complex to be stable. Two and three-tissue models with no more than four distinct kinetic parameters, including blood volume fraction, provided the best compromise between fit quality and stability/accuracy.

**Conclusion:**

For healthy men, a three-tissue model with four kinetic parameters, similar to a heart [^11^C]-palmitate model seems the most appropriate based on model stability and its ability to describe the main [^11^C]-acetate pathways in BAT cells. Further studies are required to validate this model in women and people with metabolic disorders.

**Electronic supplementary material:**

The online version of this article (10.1186/s13550-019-0497-6) contains supplementary material, which is available to authorized users.

## Background

Excess weight is a well-known risk factor for many disorders including diabetes and heart disease [1], but long-term weight loss proves difficult for many people [2]. Attempts are often frustrated by the decrease in resting metabolic rate [3] induced by caloric restriction and loss of lean mass. That is one reason why strategies to increase metabolism, such as activation of brown adipose tissue (BAT) thermogenesis, are appealing to counter metabolic disorders [4]. However, estimates of BAT energy expenditure vary greatly between studies and it remains unclear that this tissue can significantly tip the energy balance in adult humans [5–8].

Most estimates of BAT activity are based on substrate consumption (e.g., glucose and fatty acid uptake or intracellular triglyceride depletion) [7, 9, 10] and neglect confounding factors such as intracellular storage of substrates or their release into the bloodstream [11]. A workaround would be to measure oxidative metabolism in addition to substrate utilization to assess which part of the substrate is actually converted into heat. For this purpose, our group and others study the pharmacokinetics of [^11^C]-acetate, a positron emission tomography (PET) radiotracer validated for cardiac [12] and tumor [13, 14] imaging in humans. However, the pharmacokinetic models used to quantify oxidation are different for the heart and tumors. Moreover, human BAT is quite different from the heart and tumors due to its high heterogeneity and vastly different metabolic profiles under activated and inactivated conditions. It is not clear what the ideal model is for this unique tissue.

The purpose of this study is to determine which pharmacokinetic model offers the best compromise between accuracy (i.e., describing key steps of [^11^C]-acetate metabolism by BAT) and robustness to noise for human BAT signal with and without activation of this tissue by cold exposure. The correspondence between the resulting kinetic parameters and the expected increase in oxidative metabolism in the cold condition [7] is also assessed.

## Methods

### Human data

We used data from previously analyzed cohorts [15, 16] as well as subjects from recent unpublished studies. Studies were approved by the Université de Sherbrooke human ethics committee and participants provided informed consent. A total of 20 healthy young men (aged 28 ± 6 years), 7 older overweight healthy men (aged 58 ± 3 years), and 6 men with type 2 diabetes (aged 59 ± 4 years) were considered. Two subjects with diabetes had to be excluded due to absence of cold exposure data (*n* = 1) and poor image quality not allowing compartmental modeling (*n* = 1). It was decided that the remaining diabetic subject cohort was too small and heterogeneous to provide representative data and will be discussed once future studies on diabetic subjects are complete.

Protocols for these studies have already been published. To summarize, participants underwent PET and computed tomography (CT) scans (Philips Gemini) before and after acute cold exposure. Each session consisted of a baseline period at room temperature (22–25 °C), followed by cold exposure using a liquid-conditioned suit perfused with water at 18 °C. At room temperature and at *t* = 90 min of cold exposure, a CT scan of the cervicothoracic region was performed for attenuation correction, followed by a 30-min list-mode dynamic PET of the same region with [^11^C]-acetate (~ 175 MBq bolus). Depending on the study, other radiotracers as well as pharmacological interventions may have been implemented. The scope of this work is to determine whether kinetic models can assess metabolic differences between baseline and cold conditions. Therefore, only [^11^C]-acetate data acquired at room temperature (control; “warm” condition) and at 18 °C *without* administration of pharmacologic agents (maximum BAT activation; “cold” condition) were considered.

Image reconstruction of the [^11^C]-acetate data was performed using the vendor’s 3D row action maximum likelihood algorithm with corrections for attenuation, random events, scatter, and decay. Time frames were 24 × 10 s, 12 × 30 s, 4 × 5 min.

**Fig. 1:**
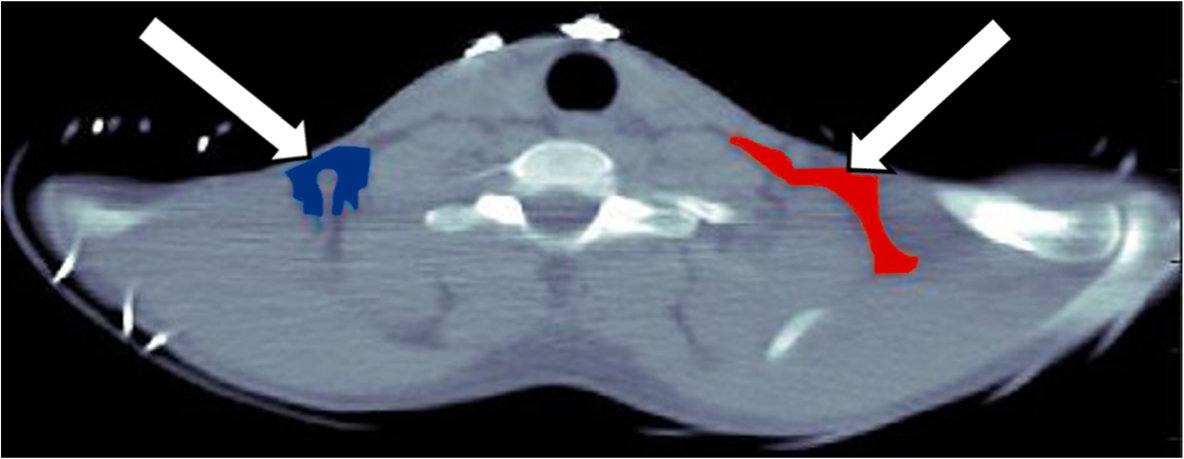
Typical regions of interest placement for brown adipose tissue based on axial CT images. These regions of interest cover the whole supraclavicular fat depot

Blood and BAT time-activity curves (TAC) were derived from regions of interest drawn on the axial CT image and reported on the registered PET image. The BAT regions of interest encompass the whole supraclavicular fat region visible on CT (Fig. 1) and vary in size depending on subject adiposity. The arterial input function (AIF) was extracted from a circular region of interest placed on the aortic arch with a diameter of ~ 2 cm. The aortic arch was selected for AIF determination because the heart is not visible on the PET-CT images centered on supraclavicular BAT. A previous study has shown that partial volume is minimal for vessels with diameters ≥ 2 cm on the Gemini scanner with [^11^C] or [^18^F]-labeled tracers [17].

Previous pharmacokinetic analyses of [^11^C]-acetate by Blondin et al. derived an index of oxidative metabolism based on a monoexponential decay model [15]. This method will be reviewed here along with more complex models.

Two-tailed paired *t* tests with a 95% confidence interval (*p* ≤ 0.05) were used to compare kinetic parameters. No correction was applied for multiple comparisons. All statistical analyses were performed with Prism (GraphPad Software).

### Monoexponential decay model

**Fig. 2:**
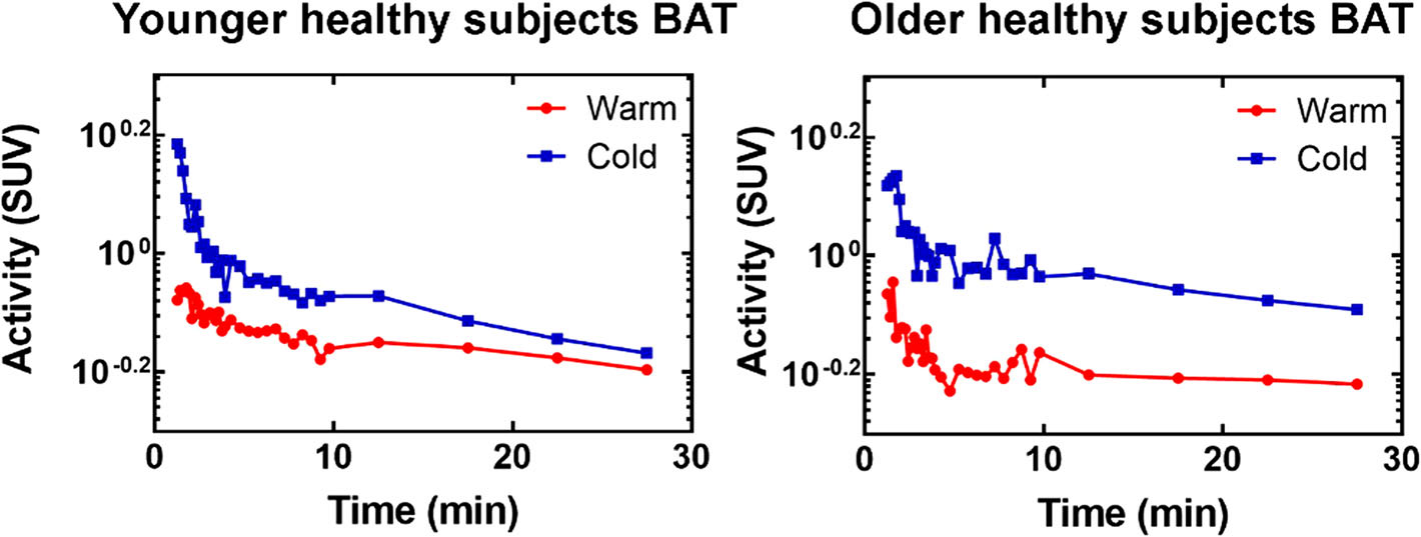
Semi-log plots of mean BAT TAC in the washout phase

The monoexponential model is a simplification of a one-tissue compartmental model that does not take into account the vascular component of the signal and the AIF. A monoexponential fit is applied to the first part of the clearance phase as suggested by Armbrecht et al. for the heart [18]. The resulting decay constant is the oxidative index, *K*_mono_. Time points corresponding to this phase are selected by looking at semi-log plots of the BAT TAC (Fig. 2). Unlike in the heart, where a clearly monoexponential decay occurs in the 5–10 min range, older subject BAT TAC show a flat profile starting around 5 min. Therefore, the time frame from 1.5 to 5 min was selected even though contribution of the blood signal is more important during this period than for later time points. Nonlinear fit was performed in Prism.

### Pharmacokinetic models

**Fig. 3:**
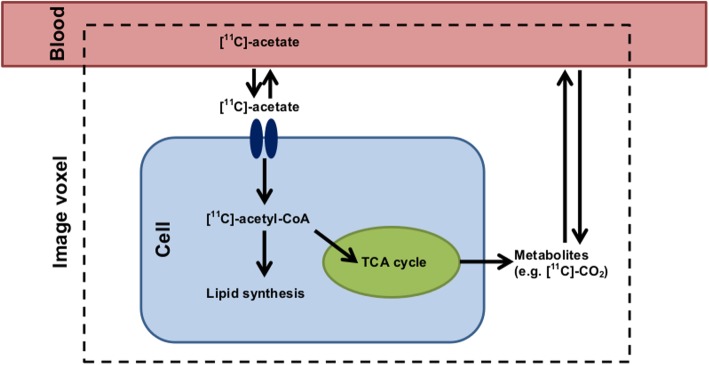
Principal pathways of [^11^C]-acetate metabolism in BAT cells. In reality, the imaging voxel (4 mm^3^) comprises many cells and blood vessels. [^11^C]-acetate exchanges rapidly through the capillary wall [18] and is transported into the cell by monocarboxylate transporters [35]. It is then converted to [^11^C]-acetyl-CoA by acetyl-CoA synthase. [^11^C]-acetyl-CoA is a substrate for both the TCA cycle (oxidation) in the mitochondrion and for fatty acid synthesis

Possible models were selected to reflect the fate of acetate in BAT cells, the surrounding blood vessels, and the extracellular space (Fig. 3). Once [^11^C]-acetate is converted to [^11^C]-acetyl-CoA in the cell, two main pathways are possible: oxidation (main pathway for the heart) and incorporation into lipids (main pathway for tumors). In BAT, it is possible that the preferential pathway changes depending on tissue activation. Inactive BAT is likely to store energy in the form of lipids, whereas active BAT produces heat through oxidation [19].

Although physiology is used to guide the selection of a pharmacokinetic model, severe limitations on model complexity are placed by the imaging modality. Notably, PET is only sensitive to the presence of [^11^C] atoms and not to their parent molecule (e.g., acetate, carbonate or lipid); therefore, species cannot be directly identified. Moreover, the only steps in acetate metabolism that can be resolved through PET are rate-limiting steps occurring on a time scale consistent with the scan temporal resolution. In other words, metabolic steps occurring faster than the ~ 5–20 s required to produce an image and those occurring slower than the 20–30 min scan duration cannot be studied. Finally, two processes occurring at the same time point with similar rates cannot be distinguished.

Based on these assumptions, the possible steps that could be modeled are (1) uptake of [^11^C]-acetate (passage from the blood to the extravascular space and/or the cell); (2) oxidative metabolism with elimination of [^11^C]-CO_2_ or other [^11^C]-labeled metabolites to the blood; (3) storage of [^11^C] as [^11^C]-acetate, [^11^C]-acetyl-CoA, TCA cycle intermediates, or lipids; (4) uptake of radiometabolites from the blood (mainly [^11^C]-carbonate); and (5) increase of the signal due to [^11^C] in the capillaries.

**Fig. 4:**
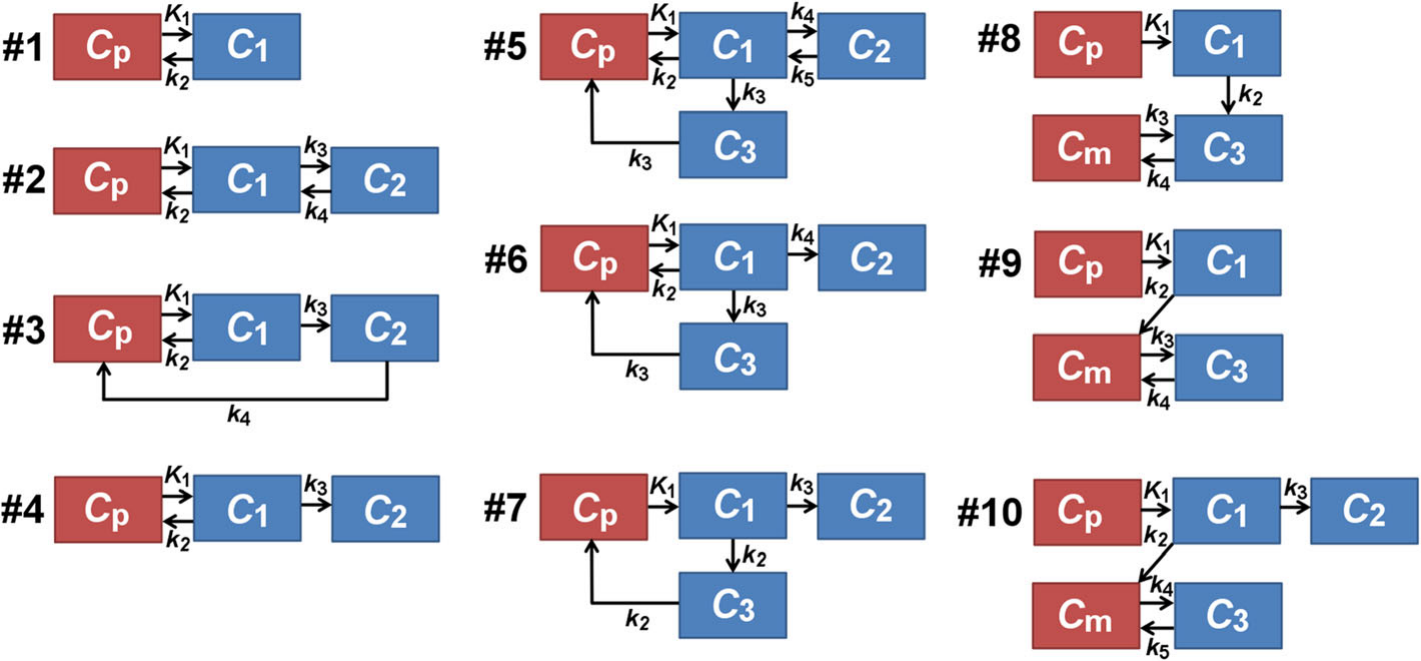
Pharmacokinetic models examined in this study. *C*_p_: plasma concentration of [^11^C]-acetate, also known as the arterial input function or AIF. *C*_1_-*C*_3_: concentration of [^11^C]-acetate or its metabolites in different physical or biochemical compartments. Tissue compartments are in blue, vascular compartments are in red. *K*_1_: flow constant (mL/g/min); *k*_2_-*k*_5_: rate constants (min^−1^). All models also include a blood volume parameter (*v*_b_) to account for the vascular [^11^C]-acetate and metabolite signals. For the four-compartmental models (#5, #6, and #7), the entry and exit rates for the oxidative compartment are set to the same value

The many different possible compartmental models are presented in Fig. 4 and are numbered from 1 to 10. They range from the simplest model (#1) representing only uptake (*K*_1_) and oxidative metabolism (*k*_2_) to the most complex model (#5) representing reversible uptake to an intracellular acetate/ acetyl-CoA pool (*K*_1_/*k*_2_), oxidative metabolism (*k*_3_), and reversible intracellular storage of metabolites (*k*_4_/*k*_5_). These models are similar to those considered by De Jong et al. for [^11^C]-palmitate [20]. Unlike [^11^C]-palmitate, [^11^C]-acetate produces a high level of circulating metabolites, mainly in the form of carbonate [12]; these species may enter BAT cells and contribute significantly to the signal. Therefore, models with a metabolite input function, similar to those proposed for [^18^F]-fluoro-DOPA in the brain [21], were also considered (models #8–10). All models include the contribution of vascular [^11^C]-acetate and metabolite signals (*v*_b_). Preliminary analyses have shown that four-compartment models are very unstable, unless the rate of entry in the oxidative compartment is the same as the rate of exit from this compartment. This constraint was included for models #5–7. More complex models proved too unstable for the current dataset and were not considered.

Model analysis was performed in MATLAB (The MathWorks). Differential equations associated with each model (e.g., Eq. 1 for model #2) were implemented using a numerical first-order differential equation solver (Runge-Kutta):

1 $$ {\displaystyle \begin{array}{c}\frac{\mathrm{d}{C}_1(t)}{\mathrm{d}t}={K_1}^{\ast }{C}_p(t)-\left({k}_2+{k}_3\right)\ast {C}_1(t)+{k_4}^{\ast }{C}_2(t)\\ {}\frac{\mathrm{d}{C}_2(t)}{\mathrm{d}t}={k}_3{C}_1(t)-{k}_4{C}_2(t)\end{array}} $$

$$ {C}_{tot}=\left(1-{v}_b\right)\left(\ {C}_1(t)+{C}_2(t)\right)+{v}_b{C}_b(t) $$

where *C*_p_ is the plasma [^11^C]-acetate concentration, *C*_b_ is the whole blood [^11^C] concentration (including metabolites), *C*_1_-*C*_2_ are the [^11^C] concentration in each compartment, *C*_tot_ is the total concentration for blood and tissue, *K*_1_ is the flow constant (mL/g/min), *k*_2_-*k*_4_ are rate constants (min^−1^), and *v*_b_ is the fractional blood volume.

Fitting the models to experimental and simulated TAC, interpolated to 1-s intervals, was achieved with a trust region reflective algorithm and uniform weighting. Initial guesses provided to the algorithm were low for *K*_1_-*k*_n_ (0.01 mL/g/min or min^−1^) and comparatively high (0.1) for *v*_b_. In previous simulations, we observed that this approach increases our likelihood of finding the global minimum in the optimisation problem for representative [^11^C]-acetate TAC shapes.

All models were first applied to human data to assess fit quality. This was performed by visual inspection of the resulting model curves and residual plots. Then the Akaike information criterion (AIC) [22] was calculated to provide an objective metric that penalizes overfitting. Models were also tested for stability, accuracy, sensitivity, and identifiability through simulations.

### Input functions

As mentioned previously, metabolites constitute an important part of the [^11^C]-acetate PET signal, especially at later time points. The AIF (representing the [^11^C]-acetate available to cells) was corrected to exclude these metabolites based on literature data [14, 23] (Eq. 2a). For models requiring a metabolite input function, the metabolite curve was derived by taking the difference between the blood signal and the AIF (Eq. 2b). Both the AIF and the blood signal were corrected for delay and dispersion occurring between the aortic arch and the BAT region.

Metabolite correction of the AIF:

2a $$ {C}_p={C}_b\ast {e}^{-0.104\left(t-0.48\right)} $$

2b $$ {C}_m={C}_b-{C}_p $$

where *C*_*p*_ is the AIF, *C*_*b*_ is the blood signal, *C*_*m*_ is the metabolite input function, and *t* is the time in minutes. Values were corrected for *t* ≥ 0.48. Uptake of [^11^C]-acetate by blood cells is considered negligible [24].

Delay between the aortic arch and the BAT was estimated by comparing the maximum initial slope of the signal from the aortic arch and the signal from a large blood vessel (subclavian or carotid artery depending on BAT location) near the BAT. Dispersion values for the [^11^C]-acetate bolus were obtained by fitting Eq. 3 to the same BAT blood vessel signal [25]:

3 $$ g(t)={C}_a(t)\otimes \left[\frac{1}{\tau }{e}^{\left(\raisebox{1ex}{$-t$}\!\left/ \!\raisebox{-1ex}{$\tau $}\right.\right)}\right] $$

where *g*(*t*) is the dispersed (BAT) blood curve, *C*_*a*_(*t*) is the undispersed (aortic arch) curve, ⊗ is the convolution operator, *t* is the time, and *τ* is the dispersion time constant. For this fit, only early time frames (< 2 min) were considered to limit contamination of the blood signal by surrounding tissues, but no partial volume correction was performed.

The delay was applied as a simple translation of *C*_*b*_ and *C*_*p*_, then the dispersion was applied using Eq. 3. Finally, *C*_*b*_ and *C*_*p*_ were fitted using an analytical model [26] to simplify computation.

### Simulations

The purpose of simulations was to eliminate models that were unstable or inaccurate when applied in ideal conditions. We define these ideal conditions as a curve without noise or with a limited amount of noise fitted with the appropriate model.

For each model and cohort, ground truth curves representing the warm and cold conditions were generated using the mean kinetic parameters and input functions of human subjects. A set of 100 random noise distributions was produced based on the average count rate of a typical ROI. Because of the high count rate, the Poisson distribution could be approximated by a Gaussian distribution. Therefore, 100 Gaussian white noise distributions (mean = 0, standard deviation = 0.4 SUV for a 10-s frame) were generated using MATLAB. These noise distributions were added to noiseless curves to produce 100 noisy curves. Noise distributions were generated only once, and all simulated curves have the same noise.

Noisy and noiseless curves were then fitted using the same algorithm as for human data. The model used to fit a curve is always the same as the model used to generate the curve. For noiseless curves, the resulting kinetic parameters were directly compared to the ground truth. For noisy curves, the average kinetic parameters were compared to the ground truth and the coefficient of variation (COV; standard deviation to mean ratio) was calculated.

A model was deemed stable if the kinetic parameters obtained by fitting the noiseless curves were close to the ground truth. A model was deemed accurate if the mean kinetic parameters obtained by fitting noisy curves were close to the ground truth and COV values were low. There is no absolute threshold for stability and accuracy in terms of deviation from the ground truth or COV. Results depend on TAC shape (kinetic parameters) and noise levels. Therefore, models were sorted from best to worse for every metric and the best four models were analyzed for sensitivity and identifiability.

### Impact of the AIF shape on the TAC

Although the AIF used in this study were always derived in the same manner, there are large variations between cohorts and between subjects. To investigate the impact of the AIF on TAC shape, we generated noiseless TAC using a fixed set of kinetic parameters for selected models (#4 and #7). TAC were generated for each individual AIF as well as for mean cohort AIF.

### Sensitivity and identifiability

Sensitivity and identifiability analyses [20] were performed to determine how models react to changes in parameters and which parameters can be independently identified.

Sensitivity to a 1% parameter variation was assessed using Eq. 4 and the mean kinetic parameters obtained for human cohorts:

4 $$ {\mathrm{Sens}}_{k_i}(t)=\frac{\delta \mathrm{TAC}(t)/\mathrm{TAC}(t)}{\delta {k}_i/{k}_i} $$

where ${\mathrm{Sens}}_{k_i}(t)$ is the sensitivity function at time *t*, *k*_*i*_ is the kinetic parameter of interest, and *δ*TAC(*t*)/TAC(*t*) is the variation of the TAC induced by the small parameter variation *δk*_*i*_/*k*_*i*_.

A sensitivity matrix was generated in the process (Eq. 5):

5 $$ {\mathrm{SM}}_{k_i{k}_j}={\int}_0^T{\mathrm{Sens}}_{k_i}\left(\tau \right){\mathrm{Sens}}_{k_j}\left(\tau \right)\mathrm{d}\tau $$

where ${\mathrm{SM}}_{k_i{k}_j}$ is the sensitivity matrix element for parameters *k*_*i*_ and *k*_*j*_ (*i* and *j* = 1:*n*) and *T* is the duration of the scan in minutes.

The sensitivity matrix was inverted and normalized to generate a matrix of the correlation coefficients (between − 1 and 1) for each combination of parameters. Correlations were considered strong for coefficients > 0.7 or < − 0.7 [27].

If a model is not sensitive to a parameter (${\mathrm{Sens}}_{k_i}$ close to 0 for all *t*), this parameter cannot be determined accurately. Therefore, optimal models must at least be sensitive to our main parameter of interest: oxidation (*k*_2_ or *k*_3_) depending on the model. Also, if two or more parameters are correlated ($\left|{\mathrm{SM}}_{k_i{k}_j}\right|\ge 0.7$ for i ≠ j), they cannot be analyzed separately.

## Results

### Human data

**Fig. 5:**
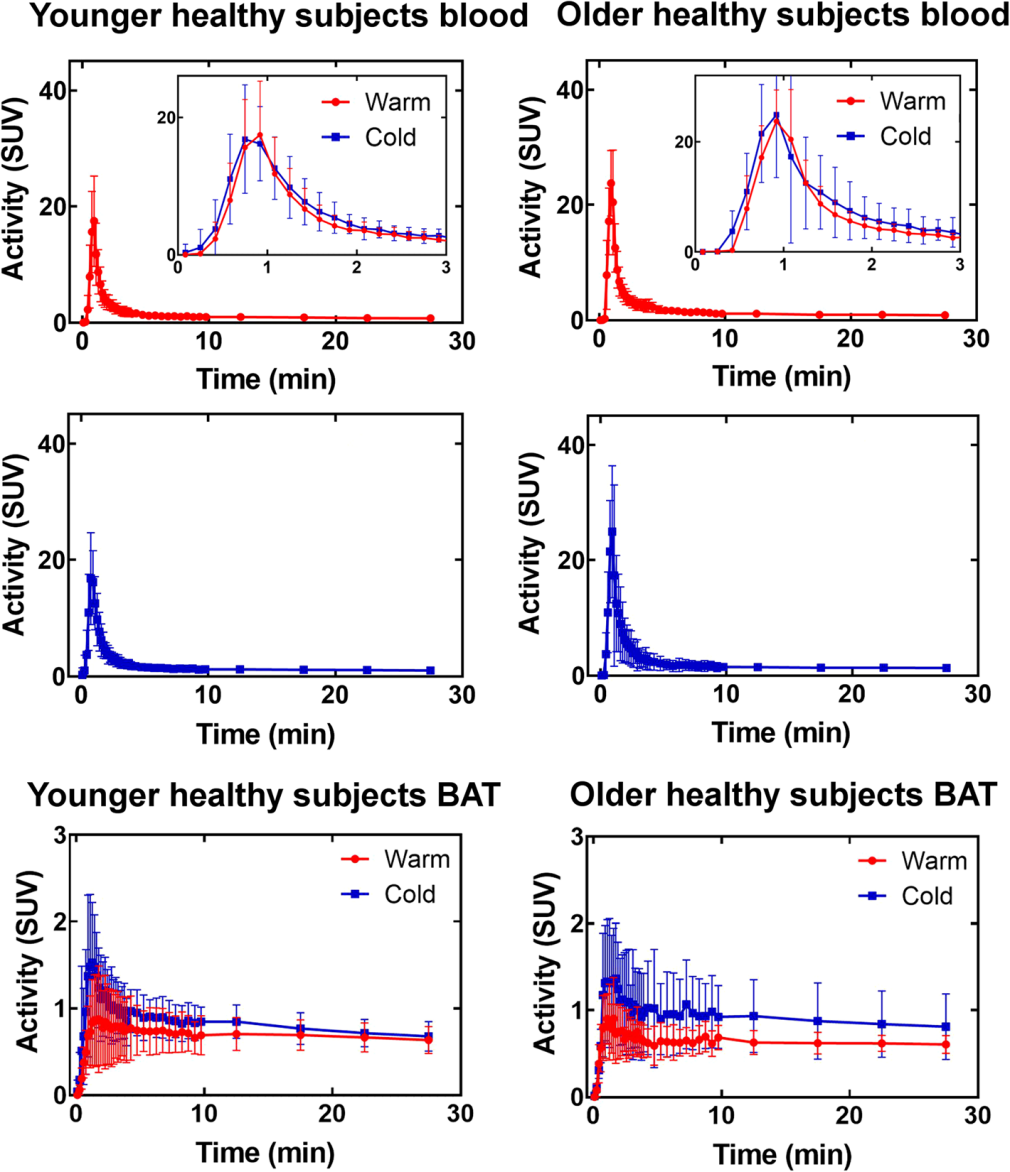
Blood and BAT activity curves. The data are shown as mean ± standard deviation for each subject cohort. The blood curve is the uncorrected signal from the aortic arch. A zoom on the blood peak signal from 0 to 5 min is shown along with the whole curve

Figure 5 shows blood and BAT curves for all cohorts in the warm and cold conditions. Even though standardized uptake value (SUV) units account for injected dose and subject weight, there are significant differences in curve shape and peak values between subjects both for blood and BAT. The blood signal generally peaks earlier in the cold condition with a somewhat slower washout. The mean area under blood curves is also different between the warm and cold conditions (Table 1). In both cohorts, this is reflected in the BAT curves. In addition, BAT signal has a higher peak in the cold. Finally, despite a lower blood signal in younger subjects, peak BAT [^11^C]-acetate activity is higher in this group for both warm and cold condition compared to older subjects.

**Table 1:** Mean area under the blood curves for different cohorts

	YS warm	YS cold	OS warm	OS cold
Area under curve (SUV)	39 ± 13	49 ± 10**	51 ± 15	63 ± 28

*YS* younger subjects, *OS* older subjects. **p ≤ 0.01 compared to YS warm

**Fig. 6:**
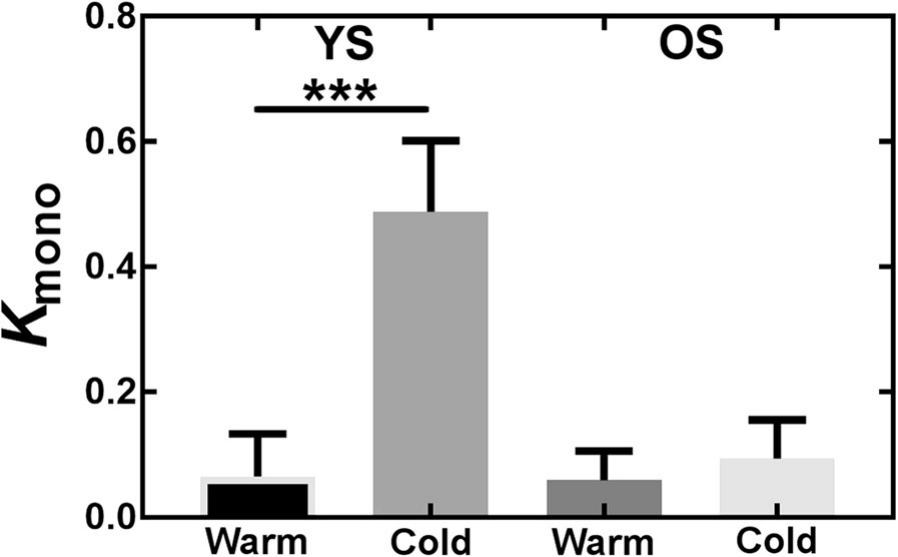
Oxidation index (*K*_mono_) from the monoexponential fit. *YS* younger subjects, *OS* older subjects. ***p ≤ 0.001

**Fig. 7:**
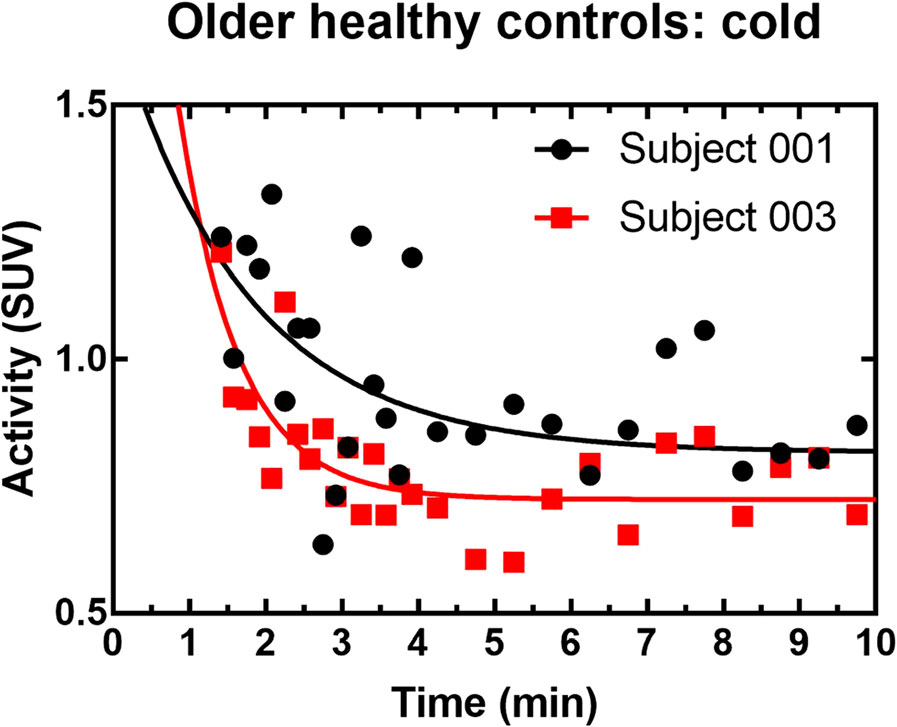
Example of monoexponential fits for representative older healthy subjects in the cold condition. The fit is performed on the portion of the curve ranging from 1.5 to 5 min but all data points in the washout phase are shown

Figure 6 shows the oxidation index, *K*_mono_. In general, fit quality is poor (Table 2), especially for healthy older subjects (Fig. 7) because TAC are relatively flat even in the early phase following the initial bolus. The mean oxidation index increases in the cold for both cohorts, but it is only significant for the younger subjects.

**Table 2:** *K*_mono_ average values and fit quality (*R*^2^)

Condition	*K*_mono_ (min^−1^)	*R* ^2^
YS warm	0.07 ± 0.07	0.15 ± 0.23
YS cold	0.5 ± 0.1***	0.79 ± 0.13
OS warm	0.06 ± 0.05	0.21 ± 0.18
OS cold	0.09 ± 0.06	0.32 ± 0.25

*YS* younger subjects, *OS* older subjects. ***p ≤ 0.001 compared with younger subjects warm condition

**Fig. 8:**
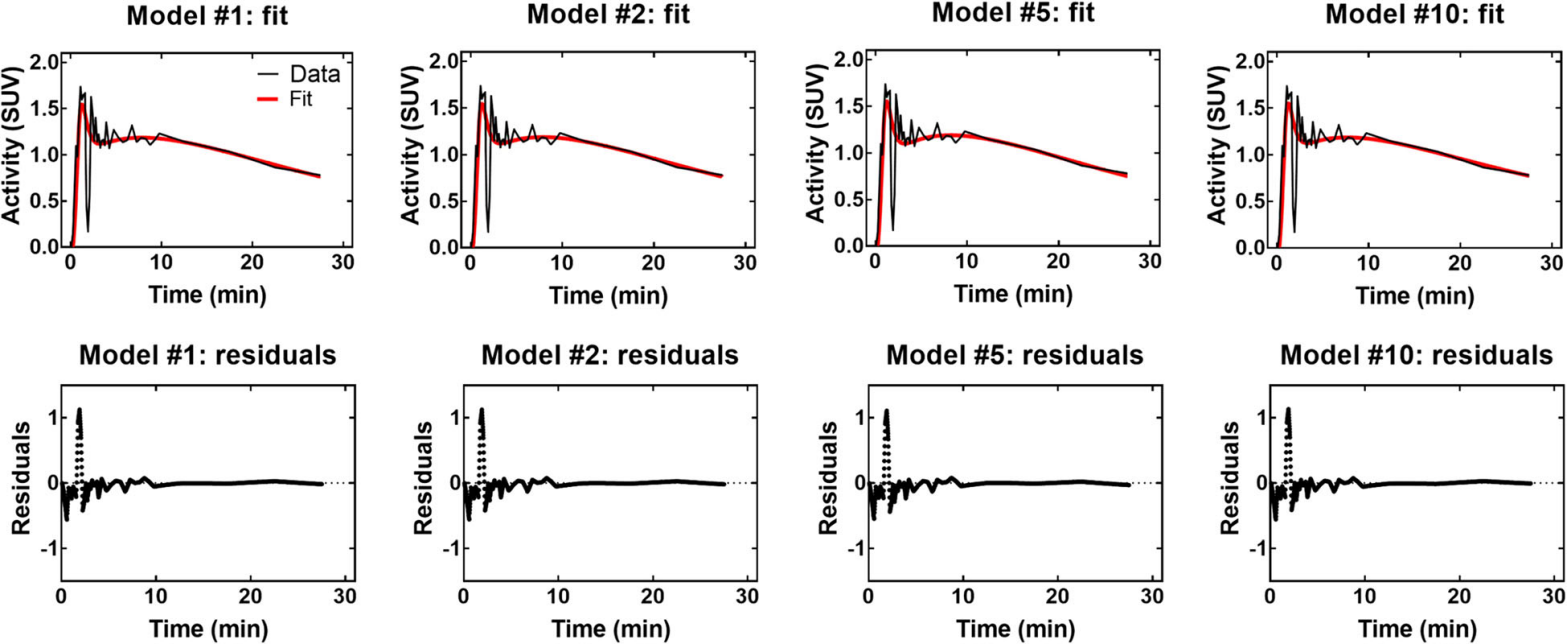
Typical fits and corresponding residual plots for selected models. These fits are for healthy younger subject #1 in the cold condition. Only one model of each type (two-compartments, three-compartments, four-compartments, and five-compartments with metabolites) is shown. Fits for other models are qualitatively similar

Figure 8 shows typical fits for selected compartmental models in one healthy young subject in the cold condition, along with the corresponding residual plots. All models are adequate to fit the initial peak of the curve and the washout slope. Residuals are generally small and randomly distributed around zero. Due to shorter initial time frames, the initial peak appears noisier than the washout slope. This noise is responsible for the higher residuals in this part of the curve. Fits of similar quality are obtained for other models, other subjects, and in the warm condition. It is impossible to determine an optimal model based on visual inspection.

Table 3 shows the mean AIC values for each model and cohort. Models give very similar results when the standard deviation is taken into account, but model #1 appears to fit data less closely than others on average.

**Table 3:** Akaike information criterion for each model

Model #	AIC warm YS	AIC cold YS	AIC warm OS	AIC cold OS
1	15 ± 12	25 ± 19	14 ± 13	25 ± 16
2	11 ± 10	23 ± 17	11 ± 15	21 ± 19
3	12 ± 9	22 ± 17	9 ± 13	21 ± 19
4	11 ± 10	23 ± 17	10 ± 12	21 ± 18
5	11 ± 10	25 ± 20	9 ± 12	22 ± 19
6	13 ± 9	23 ± 17	11 ± 13	21 ± 18
7	10 ± 9	23 ± 17	13 ± 16	22 ± 20
8	15 ± 14	30 ± 20	17 ± 18	26 ± 21
9	11 ± 9	23 ± 18	13 ± 13	22 ± 18
10	11 ± 9	23 ± 17	13 ± 11	21 ± 18

Results are shown as mean ± standard deviation. AIC values were scaled to make them positive. Lower AIC values indicate better models

*YS* younger subjects, *OS* older subjects

**Fig. 9:**
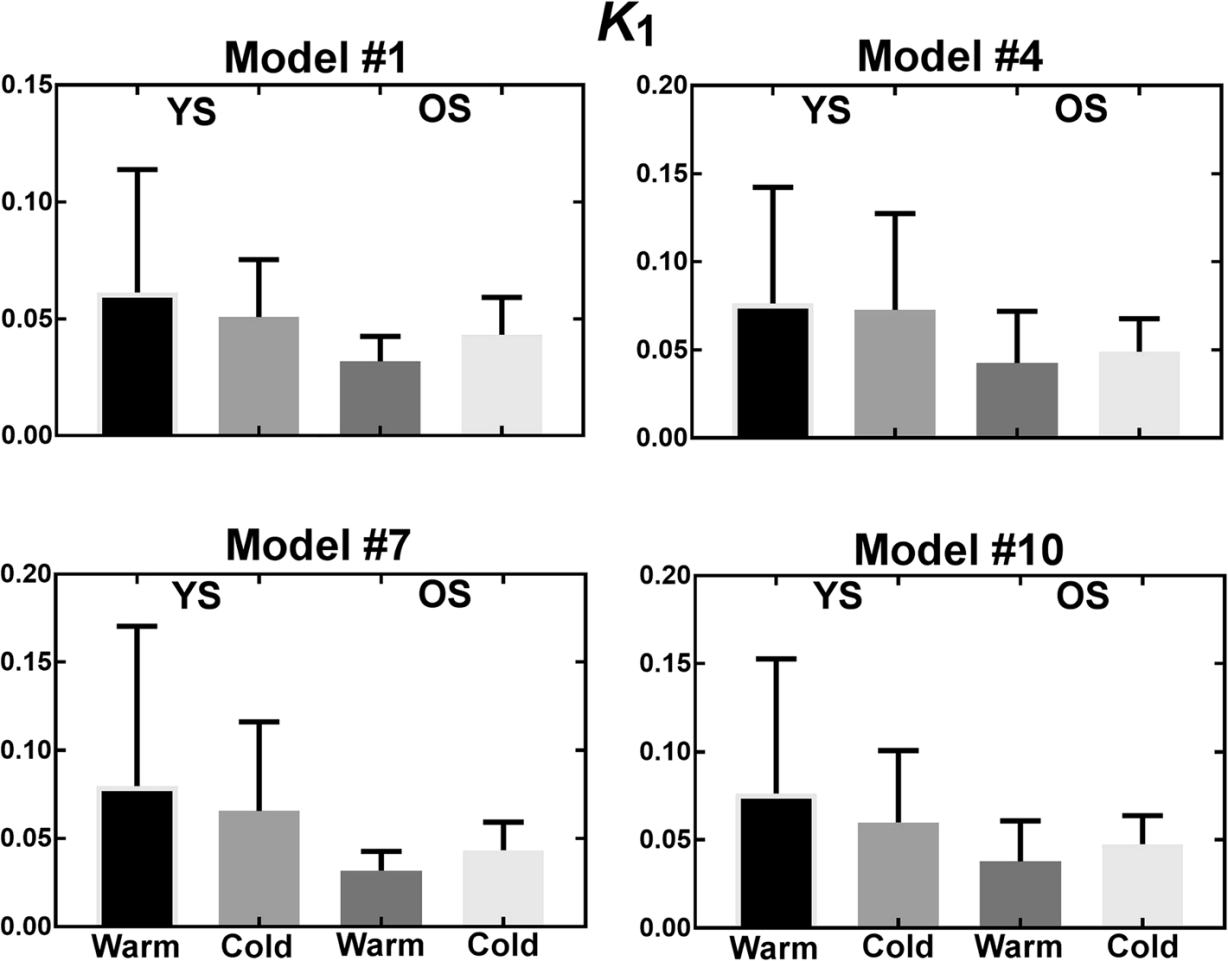
Uptake parameter for most promising models. *YS* younger subject, *OS* older subjects

**Fig. 10:**
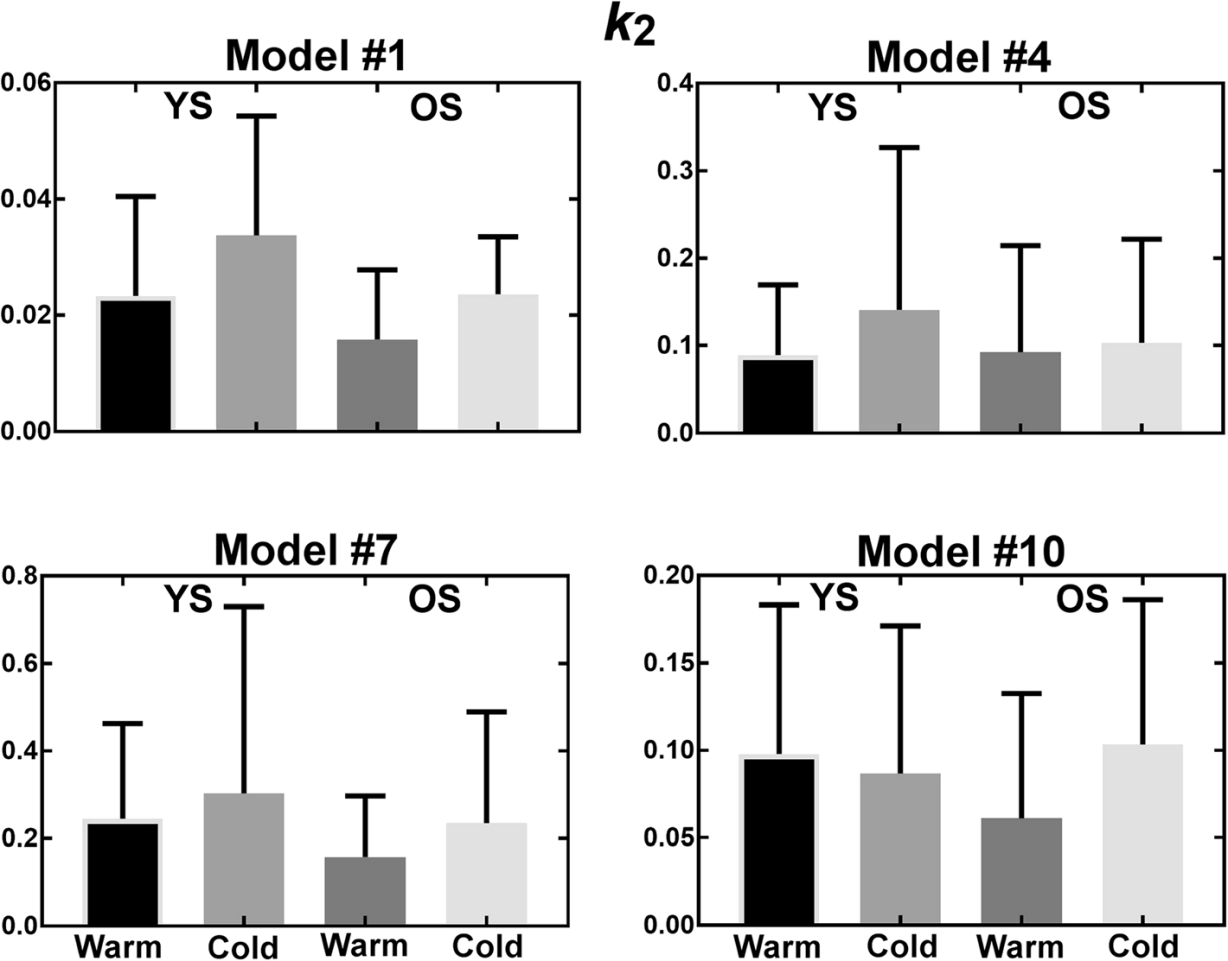
Oxidation parameter for most promising models. *YS* younger subject, *OS* older subjects

**Fig. 11:**
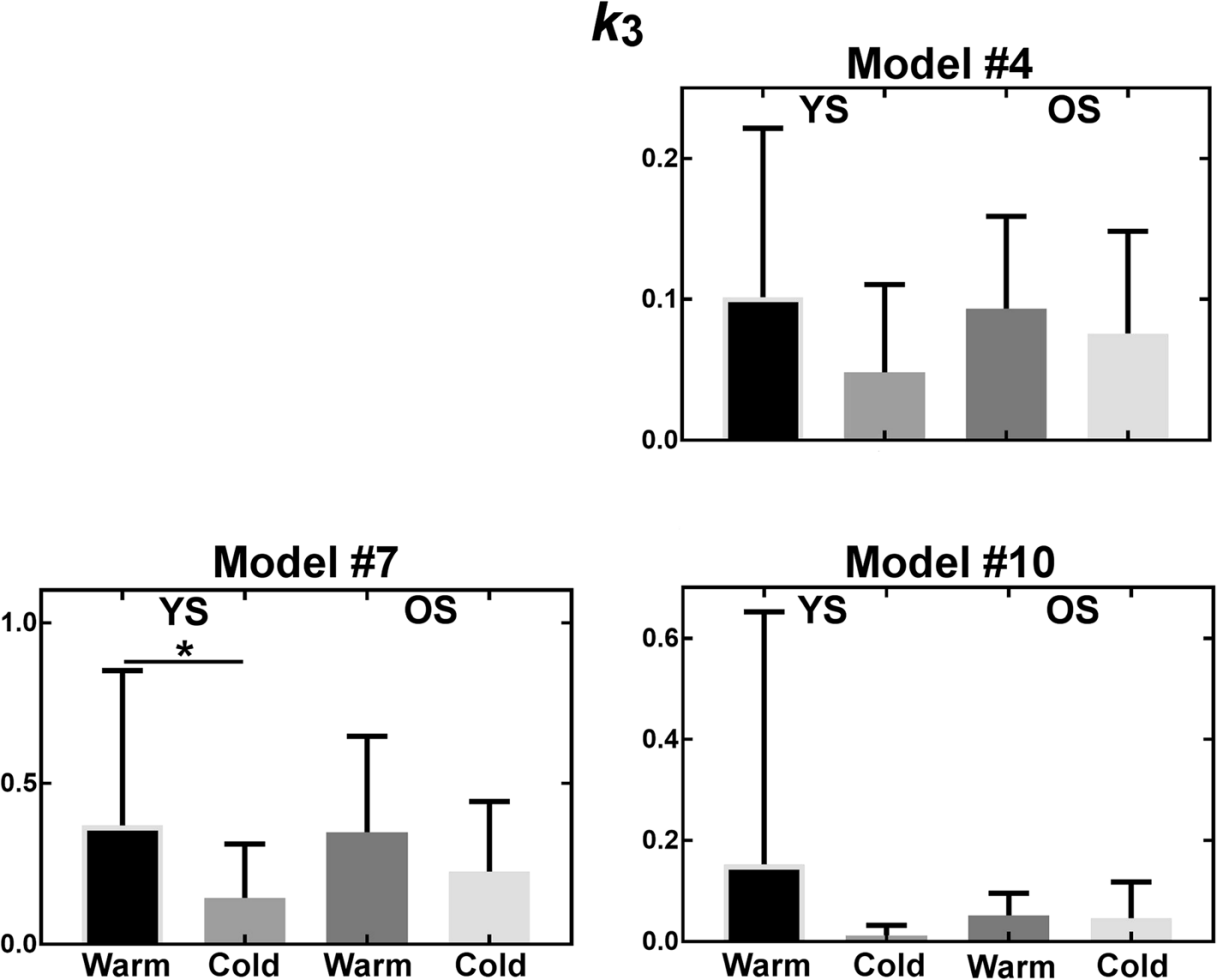
Storage parameter for most promising models. *YS* younger subject, *OS* older subjects. *p ≤ 0.05

**Fig. 12:**
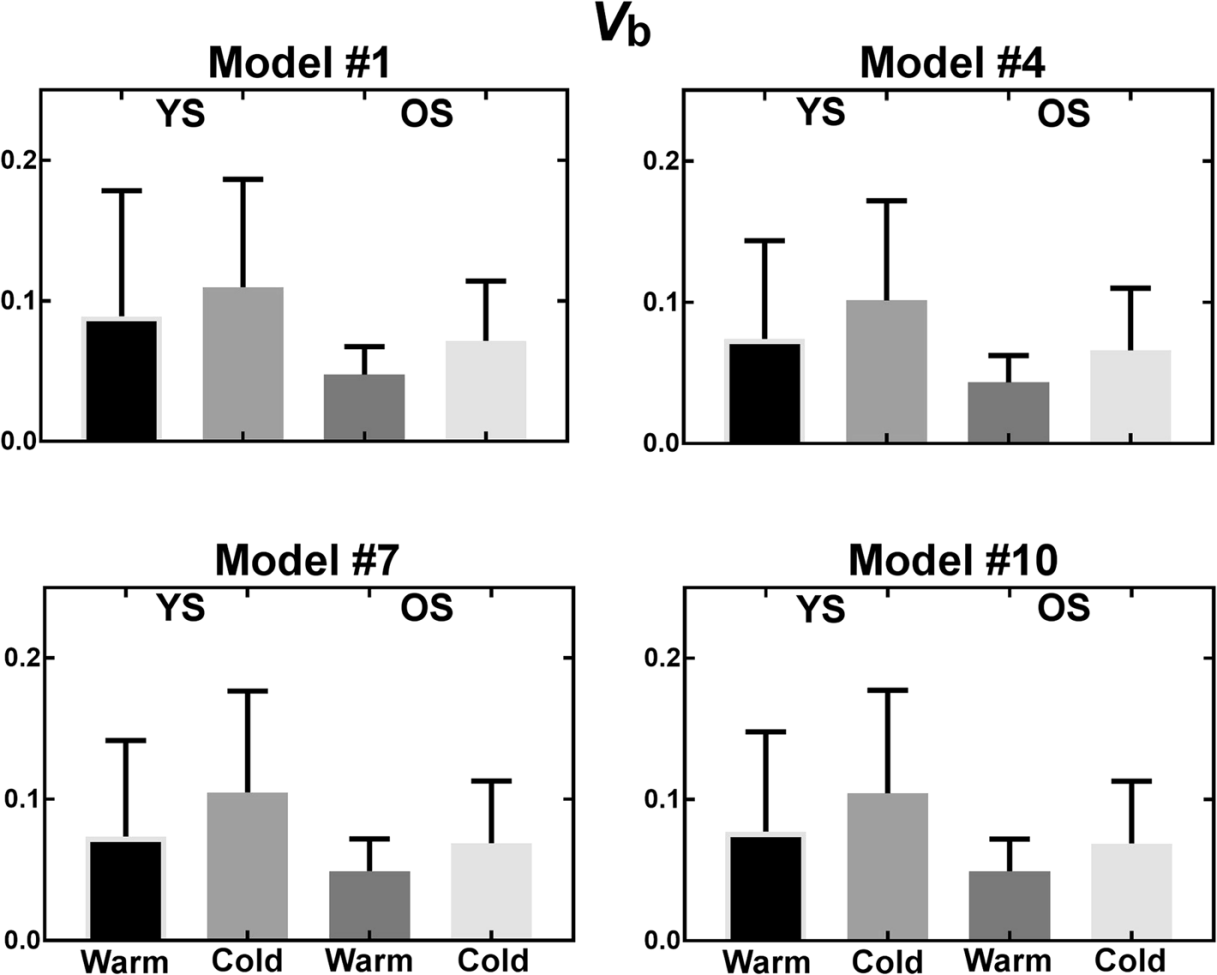
Blood volume parameter for most promising models. *YS* younger subject, *OS* older subjects

Figures 9, 10, 11, and 12 show parameters of interest for models #1, 4, 5, and 10. Note that all other models were tested (Additional file 1), but displayed models were deemed most promising based on Patlak analyses indicating that [^11^C]-acetate uptake has an irreversible component. Metabolite parameters are not shown because we deem they are not relevant to BAT activation.

As shown by the error bars in Figs. 9, 10, 11, and 12, there is important inter-subject variability with COV of 100% or more for nearly all parameters, and no model is clearly superior in terms of reduced variability. All models give similar mean perfusion (*K*_1_) and blood volume (*v*_b_) parameters for a given cohort and condition. However, the oxidation (*k*_*2*_) and storage (*k*_3_) rates are much higher for model #7 than for the other models, probably due to the extra compartment and the constraint on *k*_3_. Finally, due to the high inter-subject variability, the only significant difference is decreased storage (*k*_3_) in the cold for younger subjects (*p* ≤ 0.05) with model #7. In terms of trends, the average uptake (*K*_1_) is relatively stable in the warm and cold conditions for all models, while the average blood volume (*v*_b_) is increased. Models #1, #4, and #7 show an increase in average oxidation in the cold for younger subjects accompanied by a decrease in storage (*k*_3_). Generally, uptake (*K*_1_) and blood volume (*v*_b_) tend to be smaller in older subjects, while oxidation (*k*_2_) and storage (*k*_3_) do not show a clear trend.

Although most models show a trend toward increased oxidation and blood volume in the cold consistent with the literature [7, 28], no optimal model could be identified based on human data alone. Every model was tested in simulation.

### Simulations

**Fig. 13:**
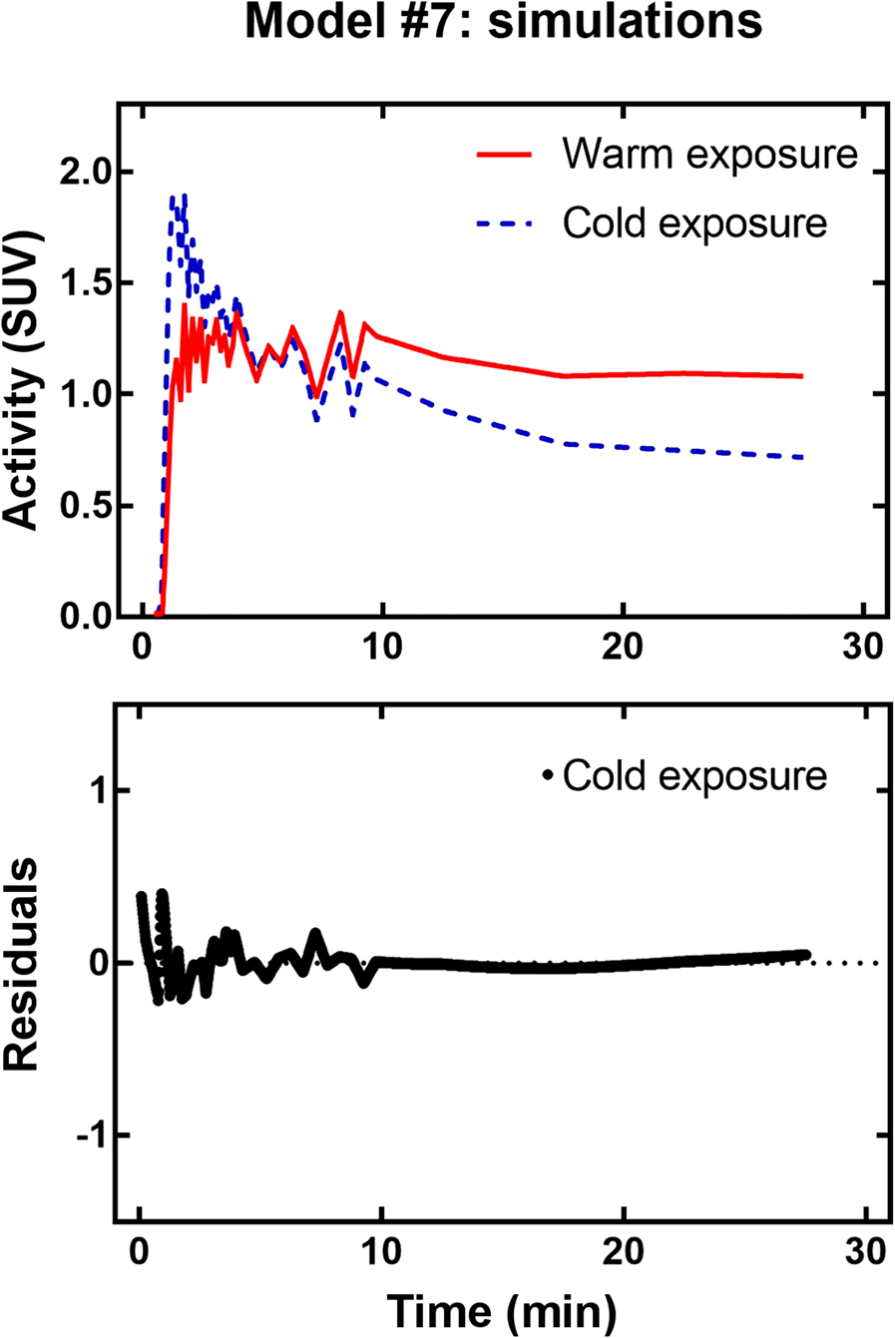
Typical curves used in simulations and typical fit residual plot

Ground truth parameters provided to the simulation algorithm were the mean parameters derived from human data. Complete results for each model are available as Additional file 2. Table 4 summarizes the results for the main parameter of interest, oxidation, and Fig. 13 shows typical noisy curves and a fit residual plot. The TAC noise pattern is reflected in the residuals, which are similar in magnitude to those obtained for human data (Fig. 8).

**Table 4:** Summary of simulation results for the oxidation parameter

Model #	Stability (%)		Average accuracy (%)		COV (%)	
Cohort	YS	OS	YS	OS	YS	OS
1	< 1*	< 1*	− 12*	− 12***	11*	17*
2	− 41	− 76	429	− 27	89	86
3	16	− 43	280	− 16	152	85
4	< 1*	< 1*	16**	8*	31***	37^***^
5	212	258	150	90	96	85
6	− 72	− 31	− 75	− 29	108	105
7	< 1*	< 1*	− 29***	− 11**	32	39
8	53	2435	163	2546	132	156
9	− 22	− 65	− 30	− 68	15^**^	64
10	− 32	− 10	− 31	− 81	53	27**

Stability: maximum percent difference between the oxidation parameter derived by fitting the noiseless curve and the ground truth. Average accuracy: maximum percent difference between the average oxidation parameter derived by fitting 100 noisy curves and the ground truth. *COV* coefficient of variation (standard deviation/mean) for 100 noisy curve fits. For each metric and each cohort, the 3 best (lowest) values are indicated (*best result, **second best, ***third best). *YS* younger subjects, *OS* older subjects

Models #1 and #7 proved the most stable and model #4 was also deemed stable. Models #1 and #4 were the most accurate with models #7, 9, and 10 providing adequate results in most cases. Note that Table 2 provides only values for the oxidation parameter, but these values reflect how models perform for other parameters.

Model #9 was rejected because it does not provide additional information compared to model #1 and is less accurate.

### Impact of the AIF shape on the TAC

The results shown here are for model #7. Similar figures can be found in Additional file 3 for model #4. Results are similar for both models.

**Fig. 14:**
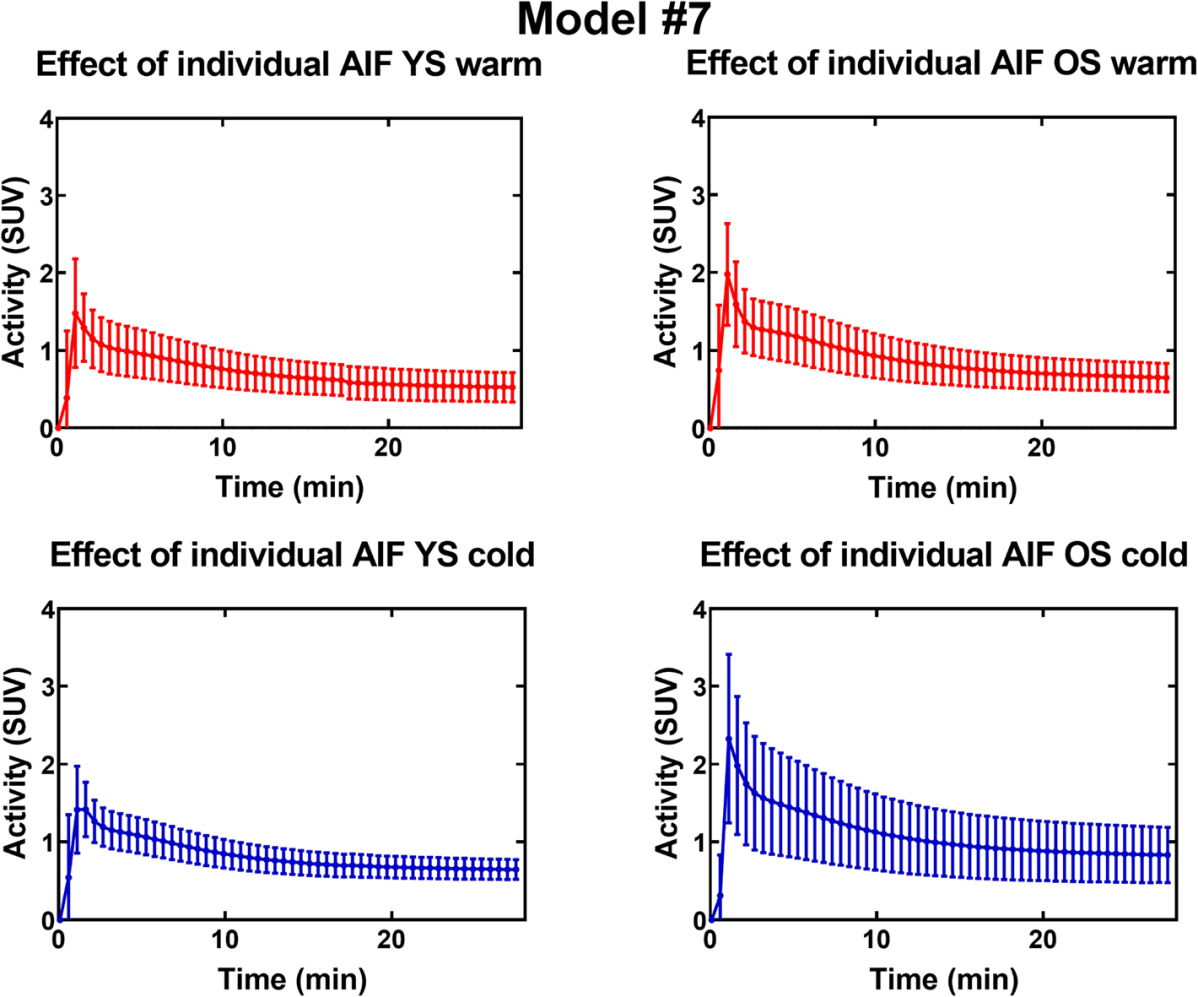
TAC variability induced by individual AIF for model #7 and set parameters: *K*_1_ = 0.07 mL/g/min, *k*_2_ = 0.30 min^−1^, *k*_3_ = 0.14 min^−1^, *v*_b_ = 0.10. *YS* younger subjects, *OS* older subjects

**Fig. 15:**
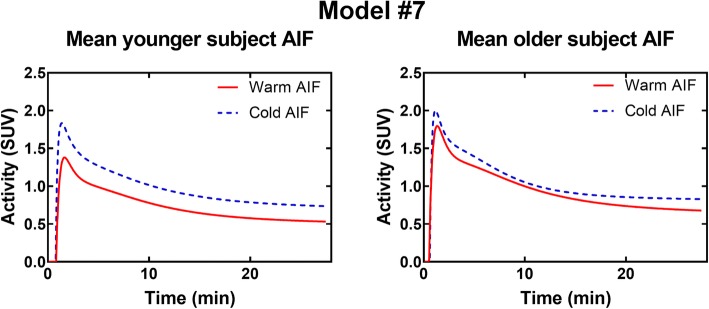
TAC generated using average AIF for model #7 and set parameters: *K*_1_ = 0.07 mL/g/min, *k*_2_ = 0.30 min^−1^, *k*_3_ = 0.14 min^−1^, *v*_b_ = 0.10

Figure 14 shows that, for a given set of parameters, there is important inter-subject variability in TAC shape (~ 20–40% on average) caused by variations in AIF. This variability is highest for older subjects in the cold condition. Differences in average AIF (i.e., the AIF used for simulations) also induce differences in TAC height (Fig. 15).

### Sensitivity and identifiability

Models #1, 4, 7, and 10 were tested for sensitivity and identifiability. Sensitivity analysis was used to visualize the impact of each parameter on the model TAC, whereas identifiability analysis was used to search for correlations between parameters. Initial kinetic parameters used for the sensitivity and identifiability analyses were the same as for previous simulations.

**Fig. 16:**
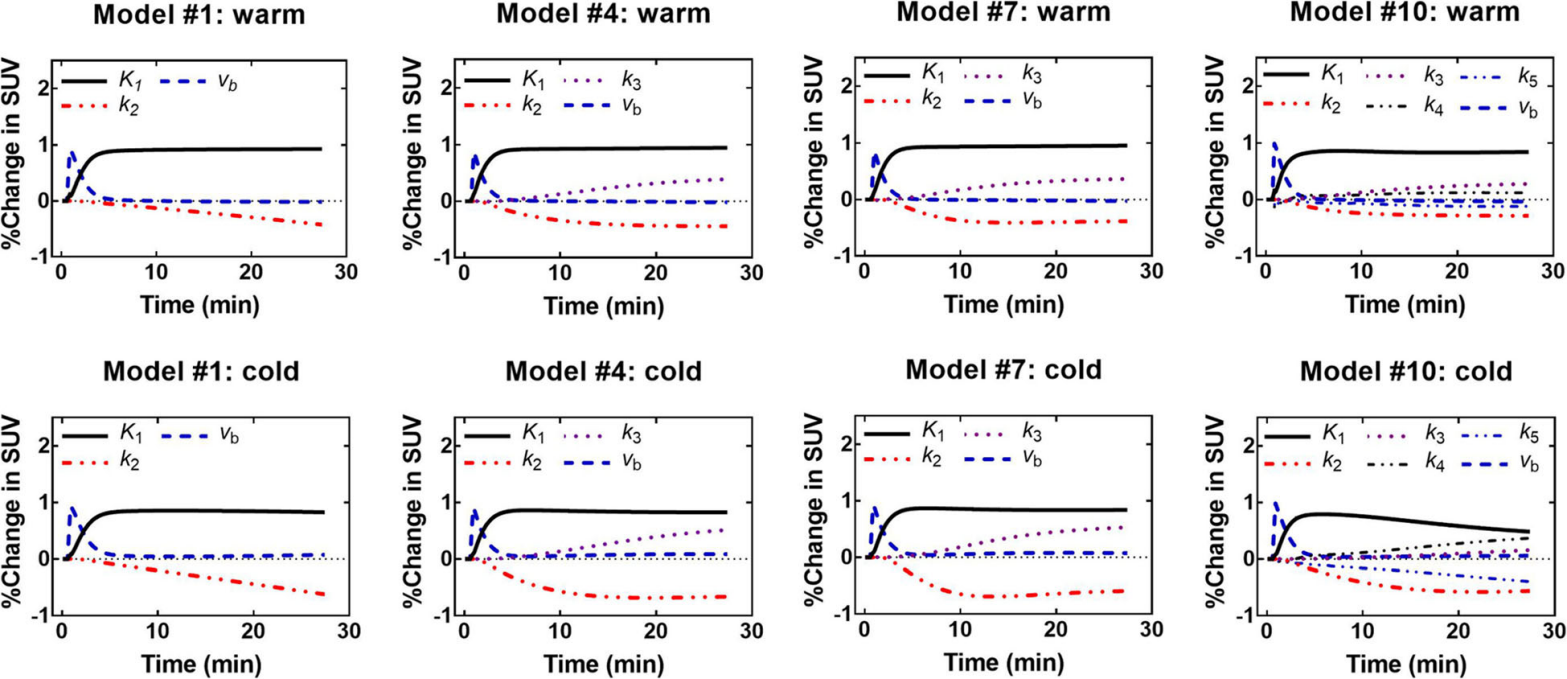
Sensitivity profiles in the warm and cold conditions for younger healthy controls

Figure 16 shows the sensitivity profiles for young controls; other sensitivity profiles are similar and available as Additional file 4. The parameters *K*_1_ and *k*_2_ have opposite effects: an increase in *K*_1_ increases the SUV values, while an increase in *k*_2_ decreases the SUV values. For *K*_1_, the maximum effect is reached after ~ 4 min; for *k*_2_, it is model-dependent. Sensitivity to *k*_3_ (storage) affects mostly the last points of the curve. Model #10 has minimal sensitivity to metabolite kinetic parameters (*k*_4_ and *k*_5_) and their effect is confined to the later part of the curve. Also, model #10 is not very sensitive to storage (*k*_3_). Finally, the *v*_b_ parameter mostly affects the first ~ 4 min of the SUV curve; therefore, it is responsible for the shape of the initial peak.

Correlation matrices have been produced for all models (Additional file 4). In most cases, *K*_1_ and *k*_2_ (uptake and oxidation) are correlated. In some cases, correlations were found between *k*_3_ (storage) and either *K*_1_ or *k*_2_. Finally, model #10 has the most correlations, notably because the metabolite parameters (*k*_4_ and *k*_5_) interact with many other parameters. The only parameter without strong correlation for all models is *v*_b_.

## Discussion

### Human data

The observed trend toward increased oxidation (*K*_mono_ or *k*_2_ depending on the model) in healthy subjects during cold exposure is in accordance with previously published data [7, 15] as is increased blood volume (*v*_b_). However, the magnitude of the *v*_b_ increase is less than suggested by [^15^O]-H_2_O PET studies [28]. Further studies comparing both radiotracers under the same conditions would be required to find the cause of this difference.

Overall, compartmental modeling seems more appropriate to assess oxidation in BAT than the monoexponential fit. Compartmental modeling uses the whole TAC instead of relying on the user to select a part of the curve to analyze, therefore reducing possible inter-user variability. Also, compartmental models account for differences in AIF and we have shown through simulations (Figs. 14 and 15) that AIF variability has a large impact on TAC shape. On the other hand, this requires validation of the AIF selection method to ensure that the differences are caused by biological factors, such as individual responses to cold and not by methodological errors.

The large inter-subject variation in BAT TAC and resulting kinetic parameters must also be pointed out. This variability is typical of BAT metabolic responses in humans and parallels measures of [^18^F]-FDG uptake [16] and radiodensity for similar cohorts [29]. It may be attributed in part to the unique biology of BAT: unlike more conventional tissues, such as the myocardium, or solid tumors, human BAT has a heterogeneous distribution (i.e., clusters of brown adipocytes are embedded among predominantly white adipocytes). The size of these clusters and the recruitment of brown adipocytes during cold exposure change with age, health status, cold acclimation, and, possibly, genetics [16, 30–32]. These specificities also pose technical challenges for PET-CT segmentation of active BAT, a further source of TAC variability.

### Simulations, sensitivity, and identifiability

No model could be excluded based on visual analysis of the fits or residual plots. Moreover, high variability in the AIC, due to high variability in TAC shapes, prevents model selection based on this criterion or other residual-based criteria. Therefore, further analyses were required to identify the most performant models. Simulations were carried out to see if a model could provide results that were both stable and accurate despite the addition of noise.

Both the complexity of the TAC and the noise level influence the maximum number of kinetic parameters that can be fitted reliably. For example, a smooth curve can be described with few parameters and adding more parameters than necessary can lead to overfitting and erroneous results even in the absence of noise. In the presence of noise, this effect is amplified as extra parameters are used by the model to fit noise-induced fluctuations.

In the present case, because curve shapes are fairly simple, only the simplest models proved stable and accurate. These models have four parameters or less, including the blood volume parameter (*v*_b_). Additional parameters in more complex models would need to be set based on a priori knowledge, for example by finding a relationship between *v*_b_ of [^15^O]-H_2_O and of [^11^C]-acetate or similar relationships for other parameters and imaging probes. We did use a priori constraints for models #5–7 because it seemed reasonable based on similar work on palmitate metabolism [20, 33]. However, we were not able to find similar constraints for uptake, oxidation, and storage.

Neglecting the *v*_b_ term would also be a way to decrease the number of parameters, but it would be ill-advised because it removes important information, such as BAT perfusion changes with activation, that can be determined precisely (*v*_b_ is identifiable and models have a high sensitivity to it). On the other hand, parameters such as *k*_2_ (oxidation) are much more variable due to correlations with other parameters such as uptake (*K*_1_). To the best of our knowledge, previous studies on [^11^C]-acetate in other tissues (e.g., the heart) did not report sensitivity profiles or correlation matrices for kinetic parameters; it certainly appears to be an important aspect to consider when interpreting changes in pharmacokinetic parameters between experimental conditions.

### Choice of an optimal model

Based on simulation results, the two-compartment heart model is the most stable and accurate for use with [^11^C]-acetate in BAT. This model is related to the monoexponential decay model used by Blondin et al. and allows assessment of oxidative metabolism while taking into account the AIF shape. However, model #1 has some of the highest AIC values for fits of human data, indicating that it fits the data less closely than other models.

On the other hand, models #4 and #7 have some of the lowest AIC values and are almost as stable as model #1. In addition, they provide information about storage of [^11^C]-acetate in the tissue, which may vary between the warm and cold conditions.

It is not clear, based on the present analyses, which model between #4 and #7 is preferable. Model #7 has higher inter-subject variability and is less accurate in simulations than model #4, but it is more stable. From a physiological standpoint (Fig. 3), it seems likely that the branching between storage and oxidation occurs when acetate is converted to acetyl-CoA which is then redirected either to an oxidative (fast) compartment or to a lipid synthesis (slow) compartment. This physiology is best described by model #7 (Fig. 4) where *C*_1_ is the acetyl-CoA synthesis compartment, *C*_2_ is the lipid synthesis compartment, and *C*_3_ is the oxidative compartment. In model #4, both conversion to acetyl-CoA and oxidation occur in the *C*_1_ compartment and storage (*C*_2_) cannot occur in parallel with oxidation.

Finally, we advise against using a model with a dual input function such as model #10 because it is not as stable as models #1, 4, and 7. Also, according to sensitivity and identifiability analyses, metabolite parameters are poorly estimated and can affect other parameters through correlations.

### Special considerations

Obtaining a precise local AIF with proper delay and dispersion is essential to fit the BAT [^11^C]-acetate TAC adequately. It was noticed early in the analysis process that the initial peak of the TAC could not be fitted properly without this correction. A simple correction based on a blood vessel signal near the BAT region provided good fit results in this work. More refined corrections methods could be attempted in order to further improve the quality of fits [34].

As mentioned previously, differences in AIF shapes between the warm and cold conditions will have to be investigated to understand how the cooling protocol impacts both the local and distal AIF. For example, activation of the sympathetic nervous system by cold exposure has effects on cardiac rhythm and blood flow, which can modify the AIF.

Finally, because the oxidation and storage parameters affects mostly the later part of the TAC (i.e., after *t* = 5–10 min), one should not limit the analysis to the early part of the TAC unless only information about blood volume is desired.

### Limitations

The older healthy subject cohort was small and composed of overweight individuals and may not be fully representative of this age group. Also, we did not have sufficient data to assess subjects with type 2 diabetes. Moreover, the study was performed on male subjects; therefore, the observations may not be valid for females. Finally, we did not address the causes of the high inter-subject variability observed in all groups.

Methodological aspects such as BAT delineation and AIF determination will have to be taken into account to ensure that differences observed are due mostly to biology. For the time being, we had to rely on image-derived AIF and metabolite estimations based on the literature. Since cold exposure may affect the metabolite fraction, direct measurement of metabolites by blood draw is required to fully validate the results presented here.

## Conclusions

Based on the present analyses and current knowledge of acetate metabolism in BAT, model #7, a four-compartment irreversible model with four free parameters seems the most appropriate because it allows full decoupling of oxidation and lipid synthesis. In addition, oxidation by BAT cannot be accurately estimated from the first 5 min of scan because the vascular effects dominate during this time period. In other words, any oxidation occurring during this time is overshadowed by [^11^C] signal from the blood pool. Moreover, because cold exposure seems to affect the AIF shape, kinetic modeling must take into account the AIF either through compartment or graphic models. Simple exponential decay models applied to the early part of the curve are likely to be contaminated by blood signal and cannot fit data as reliably as compartmental models.

Finally, by decoupling perfusion, storage, and metabolism, compartmental modeling of [^11^C]-acetate could provide a more accurate estimation of BAT energy expenditure. This may shed new light on previously acquired data as well as improve future assessment of the role of BAT in metabolic disorders and its potential as a therapeutic target.

## Additional files

Additional file 1: Results of kinetic modeling for all models and cohorts. (XLSX 98 kb)

Additional file 2: Results of simulations for all models and cohorts. (XLSX 52 kb)

Additional file 3: **Figure S1.** TAC variability induced by individual AIF for model #4 and set parameters: K1 = 0.07 mL/g/min, k2 = 0.14 min-1, k3 = 0.05 min-1, vb = 0.10. YS: younger subjects, OS: older subjects. Figure S2 TAC generated using average AIF for model #4 and set parameters: K1 = 0.07 mL/g/min, k2 = 0.14 min-1, k3 = 0.05 min-1, vb = 0.10. (PDF 257 kb)

Additional file 4: **Figure S3.** Sensitivity profiles for healthy older subjects. Sensitivity analysis was performed only for the most promising models. **Table S1.** Correlation matrices for parameters in model #1 sensitivity/specificity analysis. **Table S2.** Correlation matrices for parameters in model #4 sensitivity/specificity analysis. **Table S3.** Correlation matrices for parameters in model #7 sensitivity/specificity analysis. **Table S4.** Correlation matrices for parameters in model #10 sensitivity/specificity analysis. (PDF 274 kb)

## Abbreviations

AIC: Akaike information criterion
AIF: Arterial input function
BAT: Brown adipose tissue
COV: Coefficient of variation
CT: Computed tomography
OS: Older subject
PET: Positron emission tomography
SUV: Standardized uptake value
TAC: Time-activity curve
YS: Younger subject
