# Supplementary material for: Determination of a pharmacokinetic model for [11C]-acetate in brown adipose tissue
Source: EJNMMI Res. 2019 Mar 27;9:31. doi: 10.1186/s13550-019-0497-6 (PMC6437247; doi:10.1186/s13550-019-0497-6)
Supplement: Supplementary file 3 — Figure S1. TAC variability induced by individual AIF for model #4 and set parameters: K1 = 0.07 mL/g/min, k2 = 0.14 min-1, k3 = 0.05 min-1, vb = 0.10. YS: younger subjects, OS: older subjects. Figure S2 TAC generated using average AIF for model #4 and set parameters: K1 = 0.07 mL/g/min, k2 = 0.14 min-1, k3 = 0.05 min-1, vb = 0.10. (PDF 257 kb) [file 13550_2019_497_MOESM3_ESM.pdf]

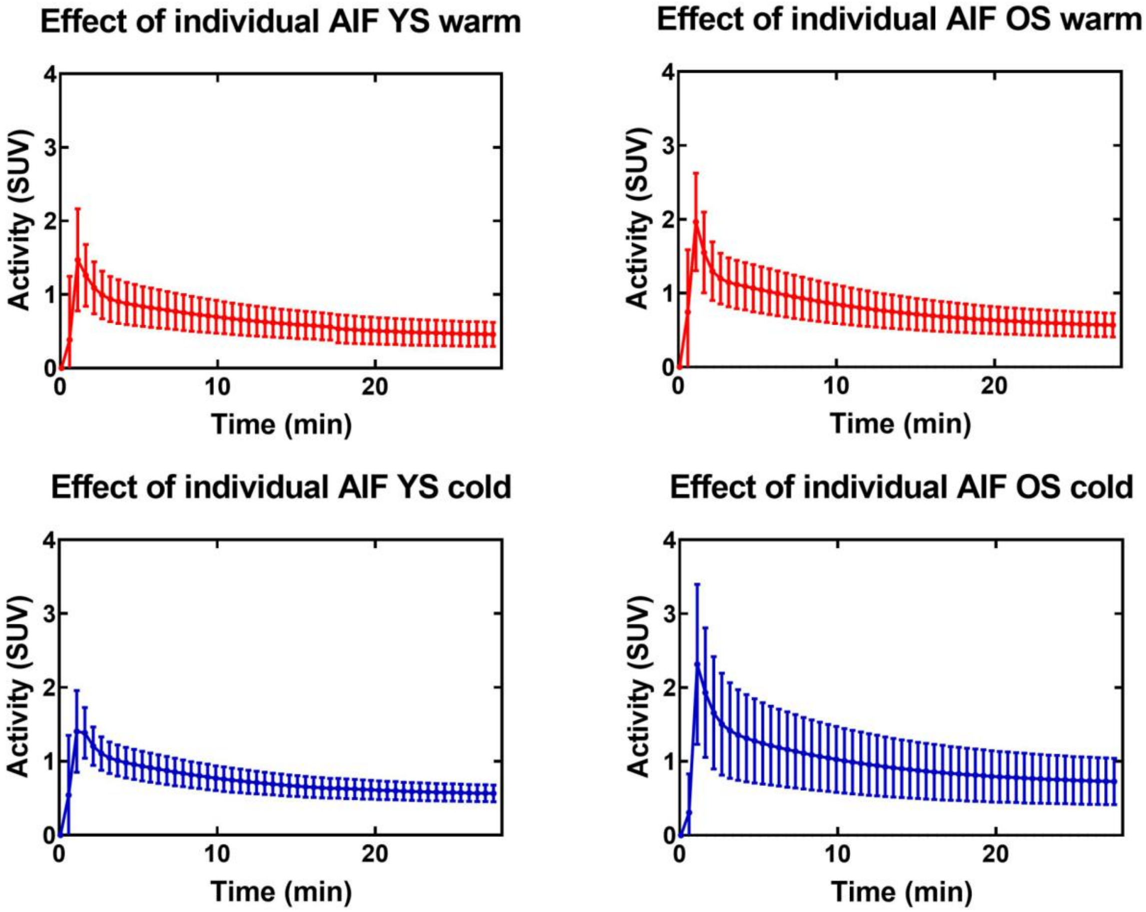

Supplementary Fig. 1 TAC variability induced by individual AIF for model #4 and set parameters: K_1=0.07 mL/g/min, k_2=0.14 min^{-1}, k_3=0.05 min^{-1}, v_b=0.10. YS: younger subjects, OS: older subjects

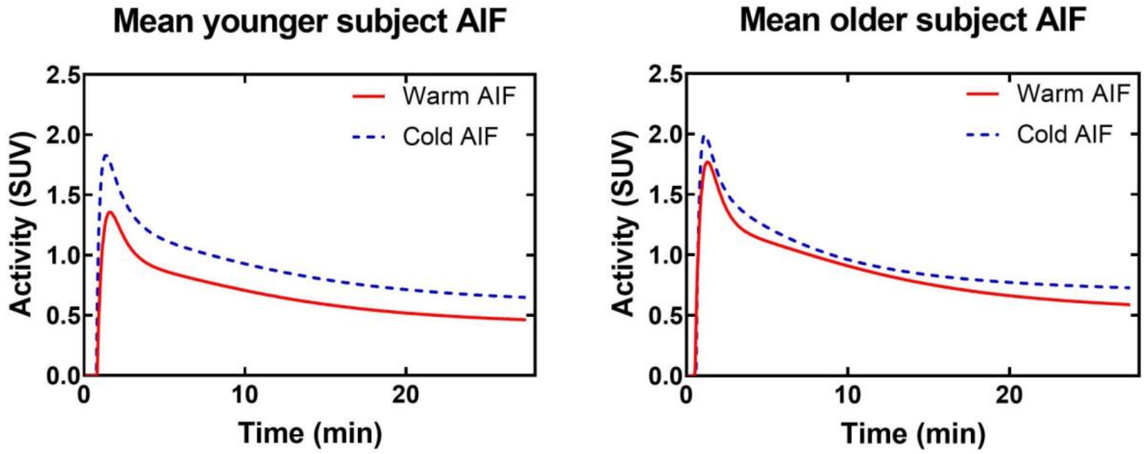

Supplementary Fig. 2 TAC generated using average AIF for model #4 and set parameters: K_1=0.07 mL/g/min, k_2=0.14 min^{-1}, k_3=0.05 min^{-1}, v_b=0.10
