# Supplementary material for: Determination of a pharmacokinetic model for [11C]-acetate in brown adipose tissue
Source: EJNMMI Res. 2019 Mar 27;9:31. doi: 10.1186/s13550-019-0497-6 (PMC6437247; doi:10.1186/s13550-019-0497-6)
Supplement: Supplementary file 4 — Figure S3. Sensitivity profiles for healthy older subjects. Sensitivity analysis was performed only for the most promising models. Table S1. Correlation matrices for parameters in model #1 sensitivity/specificity analysis. Table S2. Correlation matrices for parameters in model #4 sensitivity/specificity analysis. Table S3. Correlation matrices for parameters in model #7 sensitivity/specificity analysis. Table S4. Correlation matrices for parameters in model #10 sensitivity/specificity analysis. (PDF 274 kb) [file 13550_2019_497_MOESM4_ESM.pdf]

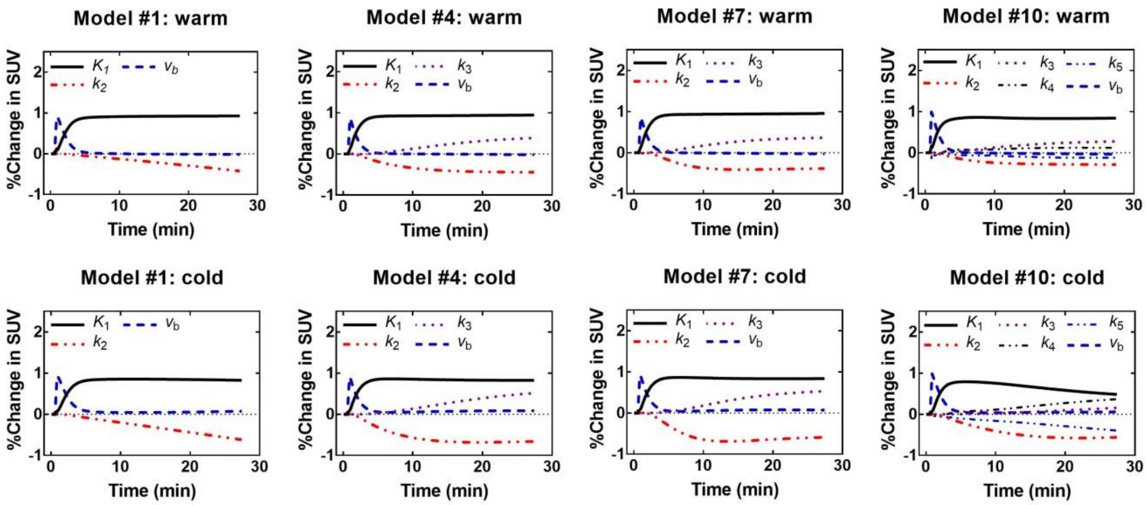

Supplementary Fig. 3 Sensitivity profiles for healthy older subjects. Sensitivity analysis was performed only for the most promising models.

Supplementary table 1 Correlation matrices for parameters in model #1 sensitivity/specificity analysis

**Warm**							
**Younger subjects**				**Older subjects**			
	K_1	k_2	v_b		K_1	k_2	v_b
K_1	1			K_1	1		
k_2	0.88	1		k_2	0.89	1	
v_b	-0.27	-0.26	1	v_b	-0.38	-0.29	1
**Cold**							
**Younger subjects**				**Older subjects**			
	K_1	k_2	v_b		K_1	k_2	v_b
K_1	1			K_1	1		
k_2	0.87	1		k_2	0.86	1	
v_b	-0.34	-0.18	1	v_b	-0.39	-0.26	1

Initial kinetic parameter values were based on human data (20 younger subjects, 7 older subjects). Strong correlations are highlighted.

Supplementary table 2 Correlation matrices for parameters in model #4 sensitivity/specificity analysis

Warm									
Younger controls					Older controls				
	K_1	k_2	k_3	v_b		K_1	k_2	k_3	v_b
K_1	1				K_1	1			
k_2	0.60	1			k_2	0.85	1		
k_3	-0.75	-0.10	1		k_3	-0.50	-0.089	1	
v_b	-0.33	-0.17	0.29	1	v_b	-0.43	-0.29	0.27	1
**Cold**									
Younger controls					Older controls				
	K_1	k_2	k_3	v_b		K_1	k_2	k_3	v_b
K_1	1				K_1	1			
k_2	0.91	1			k_2	0.92	1		
k_3	0.59	0.84	1		k_3	0.65	0.88	1	
v_b	-0.44	-0.33	-0.23	1	v_b	-0.47	-0.36	-0.23	1

Initial kinetic parameter values were based on human data (20 younger subjects, 7 older subjects). Strong correlations are highlighted.

Supplementary table 3 Correlation matrices for parameters in model #7 sensitivity/specificity analysis.

Warm									
Younger controls					Older controls				
	K_1	k_2	k_3	v_b		K_1	k_2	k_3	v_b
K_1	1				K_1	1			
k_2	0.87	1			k_2	0.90	1		
k_3	-0.82	-0.60	1		k_3	-0.98	-0.80	1	
v_b	-0.37	-0.34	0.34	1	v_b	-0.43	-0.32	0.46	1
**Cold**									
Younger controls					Older controls				
	K_1	k_2	k_3	v_b		K_1	k_2	k_3	v_b
K_1	1				K_1	1			
k_2	0.71	1			k_2	0.85	1		
k_3	-0.62	-0.076	1		k_3	0.39	0.79	1	
v_b	-0.37	-0.18	0.098	1	v_b	-0.45	-0.31	-0.12	1

Initial kinetic parameter values were based on human data (20 younger subjects, 7 older subjects). Strong correlations are highlighted.

Supplementary table 4 Correlation matrices for parameters in model #10 sensitivity/specificity analysis

Warm													
**Younger controls**							**Older controls**						
	K_1	k_2	k_3	k_4	k_5	v_b		K_1	k_2	k_3	k_4	k_5	v_b
K_1	1						K_1	1					
k_2	0.94	1					k_2	0.95	1				
k_3	0.73	0.91	1				k_3	0.83	0.94	1			
k_4	0.83	0.80	0.61	1			k_4	0.79	0.81	0.65	1		
k_5	0.046	0.051	0.028	0.26	1		k_5	0.99	0.94	0.82	0.77	1	
v_b	-0.50	-0.44	-0.29	-0.37	-0.004	1	v_b	-0.31	-0.23	-0.17	-0.19	-0.32	1
**Cold**													
**Younger controls**							**Older controls**						
	K_1	k_2	k_3	k_4	k_5	v_b		K_1	k_2	k_3	k_4	k_5	v_b
K_1	1						K_1	1					
k_2	0.10	1					k_2	0.066	1				
k_3	0.88	-0.2	1				k_3	-0.073	-1	1			
k_4	0.95	0.23	0.77	1			k_4	-0.98	0.071	-0.058	1		
k_5	0.83	-0.43	0.97	0.72	1		k_5	-0.35	-0.96	0.96	0.22	1	
v_b	-0.057	-0.38	0.15	-0.12	0.20	1	v_b	0.10	0.29	-0.31	-0.11	-0.33	1

Initial kinetic parameter values were based on human data (20 younger subjects, 7 older subjects). Strong correlations are highlighted.
